# Tularemia in the Differential Diagnosis of Lymphadenitis: A Retrospective Analysis of 16 Cases

**DOI:** 10.7759/cureus.38920

**Published:** 2023-05-12

**Authors:** Umut D Binay, Orçun Barkay, Faruk Karakeçili

**Affiliations:** 1 Department of Infectious Diseases and Clinical Microbiology, Faculty of Medicine, Erzincan Binali Yildirim University, Erzincan, TUR

**Keywords:** zoonotic diseases, waterborne diseases, tularemia, lymphadenitis, differential diagnosis

## Abstract

Introduction: Tularemia is a zoonotic disease caused by *Francisella tularensis*, a gram-negative, facultative, intracellular coccobacillus. It can occur in different clinical forms, and the most common form in our country (Turkey) is the oropharyngeal form. Unfortunately, the diagnosis of lymphadenitis caused by tularemia is delayed unless it is suspected, especially in sporadic cases. Our aim is to remind clinicians to have tularemia among differentials of lymphadenitis.

Methods: In this study, the clinical and laboratory findings of 16 tularemia patients between 2011 and 2021 were evaluated retrospectively.

Results: The mean age of the 16 patients included in the study was 39 years, and 62.5% were female. The patients were diagnosed with tularemia on the average 31st day of their complaints. The rate of use of beta-lactam group antibiotics before diagnosis was 74%. About 81.25% of the patients were engaged in animal husbandry/farming, and living in rural areas (93.75%) and farming (81.25%) were the most common possible risk factors. The patients were admitted to the hospital with the most common complaints of enlarged lymph nodes (100%), fatigue (62.5%) and loss of appetite (56.25%). All patients had lymphadenopathy, and the most common location of lymphadenopathy was the cervical region (81.25%). Moxifloxacin (56.25%) was used most frequently in the treatment of tularemia, and surgical drainage was performed for 31% of the patients.

Conclusion: The diagnosis of tularemia is often delayed unless clinical suspicion is high. Delayed diagnosis may lead to unnecessary frequent use of antibiotics, especially beta-lactam group antibiotics. As the diagnosis is delayed, since lymph node suppuration is common, surgical intervention may be required. This situation can cause extra burden for both patients and the health system. It may be beneficial to organize trainings to increase awareness among physicians and society in order to make the diagnosis early.

## Introduction

Tularemia is a zoonotic disease caused by *Francisella tularensis*, a gram-negative, facultative, intracellular coccobacillus. The disease can be transmitted to humans by the consumption of contaminated water or food, by the bite of arthropods, by direct contact with infected animals or by aerosolization [1,2]. The disease continues to be endemic in parts of North America, Europe and Asia [3]. It has also caused epidemics in different regions in different years in our country, and it can still be seen as sporadic cases from time to time or as regional epidemics [4-6]. It was most common in our country (Turkey) in 2011, and the number of cases was reported as 2,151. This number has since decreased. According to the data of the General Directorate of Public Health, 476 cases were reported in 2017 [7]. Although it is most common in the Black Sea and Marmara regions in our country, it should be noted that cases can be seen in every region of the country [7,8].

Tularemia can present in different clinical forms, and the most common form in our country is the oropharyngeal form [9]. Unfortunately, the diagnosis of lymphadenitis is delayed unless it comes to mind, especially in sporadic cases. In this study, clinical and laboratory findings of 16 tularemia patients we followed up between 2011 and 2021 were evaluated retrospectively.

## Materials and methods

Tularemia patients are followed up routinely in our clinic. The diagnosis of patients is made by serological tests. Specific agglutinins developed against *F. tularensis* lipopolysaccharide were investigated by microagglutination test and results of 1/160 and above were accepted as positive. Follow-up files are recorded for the patients. Accordingly, all tularemia patients diagnosed serologically between January 2011 and December 2021 were included in the study. Patients with an uncertain diagnosis of tularemia were not included in the study. Demographic characteristics, clinical and laboratory data of the patients were obtained from patient follow-up records. Local ethics committee approval was obtained for the study (Erzincan Binali Yildirim University, Clinical Research Ethics Committee, Date: October 27, 2022/Decision No: 04/2). Informed consent was not obtained as it was a retrospective study.

Statistical analysis

Number Cruncher Statistical System (NCSS) 2007 (Kaysville, Utah, USA) program was used for statistical analysis. Descriptive statistical methods (mean, median, frequency, ratio, minimum, and maximum) were used while evaluating the study data.

## Results

The mean age of the 16 patients included in the study was 39.43 years, and the female sex ratio was 62.5%. The patients were diagnosed with tularemia on the average 31st day of their complaints. The rate of use of beta-lactam group antibiotics before the diagnosis of tularemia was determined as 74%. About 81.25% of the patients were engaged in animal husbandry/farming, and living in a rural area (93.75%) and farming (81.25%) were the most common possible risk factors (Table 1). There was no additional comorbidity in the patient population.

**Table 1:** Demographic Characteristics of the Patients and Risk Factors for Possible Route of Transmission

Age average (min-max)	39.43 (24-68)
Gender	n (%)
Female	10 (62.5)
Male	6 (37.5)
Average day of complaints (min-max)	31.12 (3-68)
Antibiotics (used before)	n (%)
Amoxicillin clavulanic acid	10 (43.5)
Cefuroxime sodium	3 (13.1)
Ceftriaxone	4 (17.4)
Clindamycin	5 (21.7)
Ornidazole	1 (4.3)
Job	n (%)
Husbandry	12 (75)
Farmer	1 (6.25)
Housewife	2 (12.5)
Other	1 (6.25)
Possible risk factors	n (%)
Living in the countryside	15 (93.75)
Farming	13 (81.25)
Presence of similar disease in household	3 (18.75)
Presence of similar disease in village/neighborhood people	12 (75)
Presence of unprotected contact with wild animals	2 (12.5)
Presence of rodents around the house	2 (12.5)
Presence of contact with rodent	2 (12.5)
Presence of tick bite	1 (6.25)
Presence of activity in nature	6 (37.5)
Water source	n (%)
Tap water	12 (75)
Well water	4 (25)
Spring water	4 (25)
Village fountain	5 (31.25)
Lake/creek water	3 (18.75)

The patients were admitted to the hospital with the most common complaints of enlarged lymph nodes (100%), fatigue (62.5%) and loss of appetite (56.25%) (Table 2).

**Table 2:** Symptoms on Admission to the Hospital

Symptoms	n (%)
Sore throat	5 (31.25)
Sore in mouth	3 (18.75)
Weakness	10 (62.5)
High fever (>101^o^F)	7 (43.75)
Muscle-joint pains	6 (37.5)
Anorexia	9 (56.25)
Nausea/vomiting	3 (18.75)
Abdominal pain/diarrhea	2 (12.5)
Enlargement/pain in the lymph node	16 (100)
Redness/swelling in the eyes	2 (12.5)
Skin rash	2 (12.5)

All patients had lymphadenopathy, and the most common location was the cervical region (81.25%). Mean C-reactive protein (CRP) levels were 35.875 mg/L (Table 3).

**Table 3:** Tularemia Form on Admission to Hospital, Clinical and Laboratory Findings

Tularemia form	n (%)
Oropharyngeal	13 (81.25)
Glandular	3 (18.75)
Clinical findings	n (%)
Tonsillitis	3 (18.75)
Lymphadenopathy	16 (100)
Cervical	13 (81.25)
Submandibular	1 (6.25)
Axillary	2 (12.5)
Conjunctivitis	2 (12.5)
Laboratory findings	Mean (min-max)
Leukocyte count (4.000-10.000/mm3)	9,193.75 (5,800-13,100)
Neutrophil count (1800-6980/mm3)	5,926.25 (3,230-8,610)
Lymphocyte count (1000-5000/mm3)	2,450 (1,530-3,610)
Hemoglobin level (13.5-17.5 g/L)	13.375 (11-16)
Platelet count (150.000-450.000/mm3)	301,000 (191,000-336,000)
C-reactive protein level (0-5 mg/L)	35.875 (3-153)
Sedimentation rate (0-20 mg/dL)	33.06 (4-91)

Moxifloxacin (56.25%) was used most frequently in the treatment of tularemia, and surgical drainage was applied to 31.25% of the patients (Table 4).

**Table 4:** Antibiotics and Other Methods Used in Treatment

Antibiotics	n (%)
Moxifloxacin	9 (56.25)
Streptomycin	1 (6.25)
Streptomycin + Doxycycline	2 (12.5)
Streptomycin + Doxycycline + Moxifloxacin	1 (6.25)
Gentamicin + Moxifloxacin	1 (6.25)
Doxycycline	1 (6.25)
Doxycycline + Ciprofloxacin	1 (6.25)
Surgical drainage	5 (31.25)

## Discussion

Tularemia is a zoonotic disease that still maintains its importance. If its treatment is delayed, it causes morbidity, mortality and loss of workforce [3]. Especially living in rural areas, dealing with agriculture and consuming spring water are among the risk factors [10-12]. In our study, living in a rural area and being engaged in agriculture are among the possible risk factors. Consuming poorly chlorinated mains water or spring/lake/creek water is the main condition that causes infection. When the literature is examined, it is observed that the disease is more common in young women [6,13,14]. In our study, the rate of female patients (62.5%) was found to be higher. The fact that the female population living in rural areas is more exposed to spring, lake and stream waters, which is a possible transmission route, may explain this situation.

Although the most common form of tularemia in the world is the ulceroglandular form, the oropharyngeal form is the most common in our country. It is thought that this situation stems from the fact that the most common way of transmission in our country is through water sources [3-6]. In our study, the oropharyngeal form which is the most common form (81.25%) was detected, which is in line with the data of our country.

The patients most frequently presented with the complaint of swelling in the lymph nodes, and the diagnosis of tularemia is delayed unless it comes to mind. Studies have shown that patients are diagnosed late. In our study, it was determined that the average time of diagnosis of the patients was 31.12 days. In periods when the number of tularemia cases was high (it reached its peak level in 2011), the duration of diagnosis was shortened due to the large number of studies carried out by the Ministry of Health to increase awareness. However, there are delays in diagnosis, especially in regions where tularemia is not endemic, patients have to use unnecessary antibiotics due to misdiagnosis [7,11,14,15]. In our study, patients used beta-lactam group antibiotics most frequently before the diagnosis of tularemia. At the same time, delayed diagnosis causes lymph node suppuration, and approximately 32% of the patients underwent surgical drainage.

Aminoglycosides are used as the first-line therapy in the treatment of the disease, and doxycycline and quinolones are used as alternatives [3]. In the literature, it has been seen that streptomycin is used most frequently as monotherapy or in combination with doxycycline or quinolones [4,5,10,14]. In our study, it was determined that moxifloxacin was used most frequently. When the data of the patients were examined, it was observed that the need for surgical drainage did not develop in the group receiving moxifloxacin treatment. In the study by Meriç et al. [16], it was shown that treatment failure was less in patients who used quinolones and were treated earlier than 14 days. It has been stated that it will be among the new treatment alternatives [16]. In the study by Bondareva et al., which was conducted in animals, quinolones were found to be highly effective [17]. At the same time, we think that it is useful to keep in mind cases where patients cannot get an injection. However, there is a need for studies involving a large number of patients whose efficacy is evaluated. The limitation of the study is the small number of patients. There is a need for new studies in which large numbers of patients are included and treatment options are compared.

## Conclusions

The diagnosis of tularemia is unfortunately delayed unless it comes to mind. Delayed diagnosis may lead to unnecessary frequent use of antibiotics, especially beta-lactam group antibiotics. Again, as the diagnosis is delayed, since lymph node suppuration is common, surgical intervention may be required. This situation can cause extra burden for both patients and the health system. It may be beneficial to organize trainings to increase awareness among physicians and society in order to make the diagnosis early.
